# Case Report: The case of atypical OAPS complicated by positive anti-SSA and anti-SSB antibodies

**DOI:** 10.3389/fimmu.2025.1631323

**Published:** 2025-12-10

**Authors:** Chunyan Huang, Daorong Hong, Xiaoqing Chen

**Affiliations:** 1 Department of General Practice, The Second Affiliated Hospital of Fujian Medical University, Quanzhou, Fujian, China; 2 Department of Ultrasonography, The Second Affiliated Hospital of Fujian Medical University, Quanzhou, Fujian, China

**Keywords:** pathological pregnancy, antiphospholipid antibodies, anti-SSA and anti-SSB positivity, subchorionic hematoma, low-molecular-weight heparin

## Abstract

Antiphospholipid syndrome (APS) is a systemic autoimmune disease. When pathological pregnancy manifestations—such as recurrent miscarriage, stillbirth, preeclampsia, and preterm birth—predominate, the condition is called obstetric APS (OAPS). OAPS can be typical or atypical. Pregnant women positive for anti-Ro/SSA and SSB antibodies may develop fetal congenital heart block (CHB). Meanwhile, antiphospholipid antibodies (aPL) can cause preeclampsia, fetal growth restriction, intrauterine fetal death, and other adverse outcomes through mechanisms including disruption of trophoblast function. Here, we describe a case of atypical OAPS complicated with anti-Ro/SSA and SSB antibody positivity during pregnancy, who developed a subchorionic hematoma. At presentation, the hematoma was progressively enlarging, yet the patient had only one moderate-to-high titer of anti-β2-GP1 antibody and a transient low titer of ACL-IgM, not meeting clinical or laboratory criteria for OAPS. Delaying diagnosis and treatment to wait for repeat aPL testing after 12 weeks would increase risk of poor outcomes. Facing overtreatment and undertreatment challenges, we chose high-dose corticosteroids, hydroxychloroquine, and intravenous immunoglobulin therapy. When the hematoma shrank, low-molecular-weight heparin (LMWH) and aspirin were added with close monitoring, and after two weeks, both the hematoma and placental blood sinuses resolved. During pregnancy follow-up, multiple anti-β2-GP1 antibody tests showed moderate-to-high titers (over 12 weeks apart), confirming our diagnosis. Besides these immunological abnormalities, the patient was also positive for anti-SSA and anti-SSB antibodies and had a prior pregnancy complicated by suspected fetal ventricular septal defect, a high-risk factor. In light of this risk, the patient was treated with hydroxychloroquine. As a preventive strategy, two courses of intravenous immunoglobulin (IVIG) were also initiated at 16 weeks of gestation to support maternal-fetal immune tolerance. After treatment, no fetal cardiac abnormalities were observed, and a healthy female infant was vaginally delivered at 38 + 4 weeks. Diagnosing such cases and correct timing and choice of medication are critical for women of childbearing age. Most women with immune-related antibody positivity can achieve successful pregnancy and delivery with appropriate treatment.

## Introduction

Antiphospholipid syndrome (APS) is a non-inflammatory autoimmune disease characterized by clinical manifestations such as arterial and venous thrombosis and pathological pregnancy, with persistent positivity for antiphospholipid antibodies (aPL) in the serum. aPL includes anticardiolipin antibodies (ACA), anti-beta-2 glycoprotein 1 antibodies (anti-β2 GPI Ab), and lupus anticoagulant (LAC) (1). Obstetric APS (OAPS) refers to APS that primarily manifests as pathological pregnancy. According to the recommendations from the 2006 International Consensus Statement, cases that fully meet the diagnostic criteria are categorized as classic OAPS, as specified in **Table 1**. However, if only one clinical criterion or one laboratory criterion is met, the guidelines recommend diagnosing the case as atypical OAPS (1). Approximately 2% of pregnant women positive for anti-Ro/SSA and anti-SSB antibodies may experience immune-mediated fetal congenital heart block (CHB). Additionally, the recurrence rate of CHB in subsequent pregnancies is as high as 12–25% if the first pregnancy was affected (2). APS is a common cause of adverse pregnancy outcomes and has increasingly drawn the attention of obstetric clinicians, especially when adverse outcomes result from multiple immunological abnormalities. Here, we report the clinical case of one patient treated in our hospital.

**Table 1:** Revised diagnostic criteria for APS at the 2006 Sydney International APS conference.

Clinical criteria:
1. Thrombosis: Thrombosis in any organ/tissue, occurring once or more in arteries, veins, or small blood vessels (superficial venous thrombosis is not considered a diagnostic indicator). Objective evidence must be provided (e.g., imaging, histopathology). If thrombosis is confirmed by histopathology, the vessel wall at the thrombotic site must show no evidence of vasculitis.
2. Pregnancy Morbidity: (1) One or more unexplained fetal deaths ≥10 weeks gestation, with normal morphology, confirmed by ultrasound or direct examination of the fetus showing normal fetal morphology. (2) One or more preterm births of morphologically normal newborns before 34 weeks gestation due to severe preeclampsia, severe preeclampsia with severe features, or significant placental insufficiency. (3) Three or more unexplained spontaneous abortions at <10 weeks gestation, excluding maternal reproductive system anatomical abnormalities, hormonal imbalances, or genetic factors such as maternal or paternal chromosomal abnormalities.
Laboratory criteria:
1. Positive Lupus Anticoagulant in Plasma: Tested according to the guidelines set by the International Society on Thrombosis and Hemostasis (ISTH) Subcommittee on Lupus Anticoagulants/Phospholipid-Dependent Antibodies.
2. Detection of Anticardiolipin Antibodies in Serum or Plasma using Standardized ELISA: High titer positivity for IgG/IgM anticardiolipin antibodies (IgG or IgM >40 GPL or MPL, or >99th percentile).
3. Detection of Anti-β2-Glycoprotein I Antibodies in Serum or Plasma using Standardized ELISA: Positivity for IgG/IgM antibodies (titer >99th percentile of the healthy population).

ELISA refers to Enzyme-Linked Immunosorbent Assay. The above tests require a gap of at least 12 weeks, with at least two positive results. If the results of anticardiolipin antibodies are positive within <12 weeks of clinical manifestation or after >5 years, the diagnosis of antiphospholipid syndrome cannot be made.

## Case presentation

### Informed consent statement

Written informed consent was obtained from the patient for the publication of this case report and any accompanying data.

A 33-year-old female patient presented with “amenorrhea for 14 weeks and 4 days, painless vaginal bleeding for 11 days.” She had been given “dydrogesterone” for pregnancy maintenance, but the bleeding persisted. Her previous health was normal. Obstetric history: G3P1. Over a year ago, at 12 weeks and 3 days of pregnancy, she experienced vaginal bleeding. An ultrasound revealed a subchorionic hematoma, and subsequent monitoring showed multiple fetal abnormalities, including bilateral choroid plexus cysts, a suspected ventricular septal defect, and underdeveloped thorax. The pregnancy was terminated at 20 weeks due to poor prognosis. She denied any significant family history of autoimmune, thrombotic, or other relevant hereditary disorders. On admission, physical examination showed no abnormalities. Laboratory tests: normal blood and urine routine; coagulation function normal; ESR: 23mm/h; complement C3: 0.800g/L(reference range: 0.85-1.93 g/L), C4: 0.229g/L(reference range: 0.1-0.4 g/L); thyroid function tests revealed elevated anti-thyroglobulin antibodies at 3370 IU/ml(reference ≤115 IU/ml). However, thyroid function tests (TSH, FT3, FT4) were within normal limits; ANA: speckled type, 1:1000; anti-ds-DNA antibodies negative; ENA panel: anti-Ro/SSA+++, anti-Ro-52+++, anti-SSB+; ANCA: negative; ACL-IgM: 23.02 (0–18 PL/ml), ACL-IgG: normal. Obstetric ultrasound at 14 weeks and 5 days: liquid accumulations at the upper and lower edges of the placenta measuring 7.4×3.5×2.9 cm and 7.9×8.1×1.9 cm, respectively—subchorionic hematoma. MRI at 15 weeks and 2 days: extensive hematoma between the amniotic cavity and decidua, with placental margins elevated. Conservative management was continued, including hemostatic (etamsylate) and progesterone (dydrogesterone) support, in an attempt to control bleeding and maintain the pregnancy, albeit with persistent bleeding. At 16 weeks, ultrasound showed increased hematoma size with circumferential elevation of placental margins.

### Multidisciplinary consultation and diagnostic decision

Multidisciplinary consultation in our department was conducted, and the patient’s medical history was further inquired. The patient reported a history of recurrent oral ulcers but denied any rashes, joint pain, photosensitivity, hair loss, dry mouth, or dry eyes. Additional tests: rheumatoid factor negative; Coombs test positive (1+); anti-β2-GP1 antibodies: 53.51 RU/ml (0–20 RU/ml); LAC normal. Based on the multidisciplinary assessment, a diagnosis of suspected atypical obstetric antiphospholipid syndrome (OAPS) with an active autoimmune component was strongly considered. This preliminary diagnosis was primarily driven by the following findings: despite conservative treatment, the patient presented with a progressively enlarging subchorionic hematoma clinically, coupled with multiple immunological abnormalities (including high-titer ANA, positive anti-SSA/SSB antibodies, and a positive anti-β2-GP1 antibody) on laboratory investigation. A suspected diagnosis was made at this stage as the persistence of aPL positivity required subsequent confirmation. Faced with the imminent risk of pregnancy loss, a combined immunomodulatory regimen was initiated. It is critical to note that the primary goal of initiating immunosuppressive therapy at this stage was to control the presumed autoimmune-mediated process at the placental interface and prevent further hematoma expansion and potential pregnancy loss. This intervention was not based on suspected fetal cardiac involvement, for which there were no signs at that time. A basic serological workup upon admission had excluded common non-autoimmune causes of bleeding. The treatment included: high-dose methylprednisolone (40 mg/day), selected for its potent and rapid anti-inflammatory effects to quell the immune activation at the maternal-fetal interface; hydroxychloroquine (0.2 g twice daily) for its long-term benefits in APS and potential to mitigate anti-SSA/SSB-related risks; and intravenous immunoglobulin (IVIG) at 400 mg/kg/day for three days to provide broad immunomodulation and support maternal-fetal tolerance. Gastric protection and calcium supplementation were also provided. To evaluate the response to the immunomodulatory therapy, an ultrasound was performed at 17 weeks and 4 days, This scan demonstrated a marked improvement, showing a reduced hematoma: upper edge 3.1×3.2×2.3 cm, lower edge 5.5×6.5×2.7 cm; placental lesions appeared as mixed echogenic masses (5.6×4.7×3.7 cm)—likely venous lakes. Fetal heart rate remained normal. Anti-β2-GP1 antibodies rose to 111.71 RU/ml. In light of this favorable radiologic response, the methylprednisolone dose was cautiously reduced to 32 mg/day. At 18 weeks and 4 days, Doppler ultrasound showed left femoral vein thrombosis (10.1×3.2 mm). A second course of IVIG was administered, and aspirin 100 mg/day and LMWH 5000 IU/day were initiated. Steroids were tapered to 16 mg/day. A subsequent ultrasound examination at 20 weeks and 2 days confirmed the successful resolution of both the subchorionic hematoma and the placental venous lakes. At discharge (21 weeks and 6 days), anti-β2-GP1 was 59.54 RU/ml, ACL-IgM was 30.67 PL/ml, and vascular ultrasound was normal. Post-discharge treatment included methylprednisolone 8 mg/day, hydroxychloroquine 0.2 g bid, aspirin 100 mg/day, and LMWH 5000 IU/day. At 38 weeks and 4 days, she delivered a healthy female infant spontaneously, with aspirin discontinued at 36 weeks and LMWH one day before delivery.

**Figure 1:**
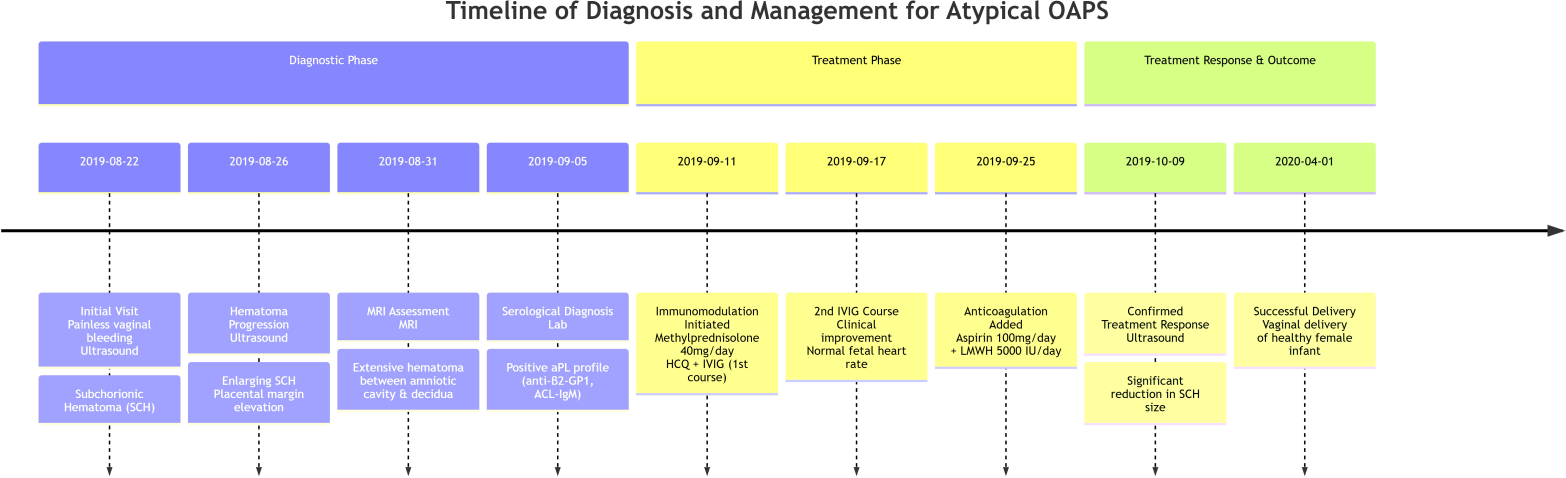
Timeline of diagnosis and management for atypical OAPS. This timeline illustrates the key clinical events, diagnostic milestones, and sequential treatment decisions. The Treatment Phase highlights the initiation of immunomodulatory therapy following serological confirmation, and the subsequent addition of anticoagulation therapy, demonstrating the rationale for the combined regimen. The favorable clinical and sonographic response supports the diagnostic impression and therapeutic approach. (SCH, subchorionic hematoma; aPL, antiphospholipid antibodies; HCQ, hydroxychloroquine; IVIG, intravenous immunoglobulin; LMWH, low molecular weight heparin.).

The patient’s complex clinical course, detailing the sequence of diagnostic findings, therapeutic interventions, and corresponding outcomes, is visually summarized in the chronological timeline (**Figure 1**).

## Discussion

The diagnosis of APS is based on the revised criteria from the 2006 Sydney International Consensus (1). In evaluating this case, the patient’s one prior full-term delivery was uncomplicated, without preeclampsia or other hypertensive disorders, which contrasts with her subsequent complicated pregnancies. Up to 10% of the general population may transiently exhibit low-titer aPL positivity (3), so overdiagnosis of OAPS should be avoided. The OAPS criteria also have limitations: they overly restrict the definition of pathological pregnancy and do not include novel aPLs. Clinically, several scenarios exist: (1) APS-like obstetric features with two positive aPL tests but <12-week interval; (2) Obstetric APS features with persistent low-titer aPL positivity; (3) Obstetric APS features with negative classic aPLs; (4) Laboratory APS criteria met but with atypical obstetric manifestations (e.g., recurrent miscarriages, unexplained late fetal loss, or severe preeclampsia with placental abruption). It is noteworthy that the American College of Rheumatology (ACR) and the European Alliance of Associations for Rheumatology (EULAR) jointly released updated classification criteria in 2023 (4). which employ a weighted scoring system. While maintaining the core elements of diagnosis, this new framework provides greater nuance and more explicitly acknowledges certain non-criteria clinical manifestations. Balancing over- and undertreatment remains a challenge, especially when the patient presents with pathological pregnancy.

When the patient presented, a subchorionic hematoma was progressively enlarging. There was only one instance of moderate-to-high titer anti-β2-GP1 antibody and one transient low titer ACL-IgM positivity, which did not meet the clinical and laboratory criteria for OAPS. Delaying diagnosis and treatment until 12 weeks later for a recheck of antiphospholipid antibodies would evidently increase the risk of adverse outcomes. Applying the aforementioned criteria to our case clearly reveals its characteristics as an ‘atypical’ or ‘non-criteria’ obstetric APS. Initially, the patient presented with only a single moderate-to-high titer anti-β2-GP1 antibody and a transient low-positive ACL-IgM. This profile failed to meet the laboratory requirement for ‘persistent positivity’ per the Sydney criteria and would also be challenging to accumulate sufficient laboratory points under the new 2023 criteria. Furthermore, her history of pregnancy termination due to fetal structural anomalies does not constitute the defined obstetric morbidity in either set of major criteria. However, the clinical presentation of this case strongly suggests an underlying APS pathophysiology. This case underscores the significant diagnostic challenges inherent in atypical presentations of OAPS, exemplified by the discrepancy between a compelling clinical presentation—a progressive, treatment-refractory subchorionic hematoma (SCH)—and the initial failure to meet strict laboratory criteria. This progressively enlarging SCH is increasingly recognized as a ‘non-criteria’ obstetric manifestation of placental vasculopathy. We addressed this dilemma through a multifaceted approach that integrated multidisciplinary assessment and accorded diagnostic weight to this salient non-criteria manifestation. The subsequent confirmation of persistently high aPL titers collectively supported an autoimmune etiology. Most compellingly, the rapid and favorable response to the combined immunomodulatory and anticoagulant regimen provided strong clinical corroboration for the diagnosis. Consequently, this case exemplifies a common clinical dilemma: a patient may not fully satisfy the formal classification criteria, yet the clinical presentation, laboratory clues, and therapeutic response strongly support the diagnosis of ‘atypical OAPS’ and necessitate proactive clinical intervention. However, the challenge in treatment lies in whether the use of low molecular weight heparin for anticoagulation and aspirin could increase the patient’s risk of bleeding. This therapeutic dilemma is further compounded by the need to accurately interpret her atypical obstetric history.

It is noteworthy that the patient’s previous pregnancy termination, due to fetal structural anomalies, does not fulfill the standard clinical criteria for obstetric APS, which primarily focuses on unexplained fetal loss or severe placental insufficiency. However, the occurrence of a significant subchorionic hematoma (SCH) in both her current and past pregnancies prompts a critical re-evaluation. A growing body of evidence suggests that antiphospholipid antibodies (aPL) can predispose to SCH formation by promoting a pro-thrombotic and pro-inflammatory state at the decidual-placental interface (5, 6). Therefore, the recurrent SCH, rather than the anomalous fetus itself, could be the key manifestation linking her obstetric history to her underlying APL positivity. This association further underscores the atypical presentation of her condition.

Subchorionic hematoma (SCH) refers to the bleeding between the chorionic plate and the decidual base, with blood accumulating in a confined space between the chorion and decidua to form a hematoma. The etiology and pathogenesis of SCH are not yet well understood. A study by Alijotas et al. (5) suggested that patients with a history of adverse pregnancy outcomes, positive autoantibodies, particularly antiphospholipid antibodies, along with low C4 and high γ-globulin levels, are more prone to developing intrauterine hematomas. Meanwhile, a higher proportion of women in the SCH group tested positive for autoantibodies, which indicates that the occurrence of SCH may be associated with autoantibodies (6). This case involves a patient with no clear history of autoimmune diseases but multiple immunological abnormalities, including ANA (speckled pattern 1:1000), positive anti-SSA and anti-SSB antibodies, transient low-titer ACL-IgM, and a one-time high-titer anti-β2-GP1 positivity. The patient experienced subchorionic hematomas in both previous pregnancies. Coagulation and liver function issues were excluded, and after obstetric pregnancy preservation and hemostatic treatment, the hematoma increased in size. The presence of autoantibodies increased platelet aggregation tendencies, leading to thrombosis or vasculitis, thereby increasing the likelihood of SCH formation. The patient was highly suspected of having atypical Antiphospholipid Syndrome (APS).Initially, high-dose steroids, hydroxychloroquine treatment, and intravenous immunoglobulin (IVIG) were chosen. As the hematoma reduced in size, LMWH and aspirin were added with close monitoring. Two weeks later, both the subchorionic hematoma and placental blood sinuses had disappeared. Subsequent follow-up throughout the pregnancy showed multiple instances of high-titer anti-β2-GP1 antibody positivity (intervals over 12 weeks), ultimately confirming our diagnosis. The use of anticoagulants and immunosuppressive drugs has been shown to have a positive effect on improving the prognosis of antiphospholipid syndrome or autoimmune diseases (7–10). Low-molecular-weight heparin has been widely used in the treatment of recurrent miscarriage. Research shows that heparin not only has an anticoagulant effect in preventing pregnancy complications in patients with autoimmune diseases but can also block complement activation.

In this case, in addition to being positive for APL, the most significant immunological abnormality is the presence of both anti-SSA and anti-SSB antibodies. Three retrospective studies suggest that hydroxychloroquine may have a potential role in preventing the development of cardiac conduction block (CHB) in pregnant women with a history of previous episodes (11–13).Furthermore, although the patient had high-titer anti-thyroglobulin antibodies, her normal thyroid function ruled out thyroid dysfunction as a primary cause of the adverse pregnancy outcome, thereby strengthening the argument that her clinical presentation was primarily driven by her abnormal antiphospholipid antibody profile. For pregnant women who test positive for anti-Ro/SSA and/or anti-La/SSB antibodies, it is recommended to perform regular echocardiographic follow-up from 16–18 weeks of gestation until delivery as a screening method for CHB. This patient had a history of suspected fetal interventricular septal defect in the previous pregnancy, which is also considered a high-risk factor. Immunoglobulin contains unique antibodies against placental trophoblast antigens and their specific targets, which help compensate for the lack of protective antibodies in women with threatened miscarriage. Additionally, it binds to natural killer (NK) cell receptors, blocking their cytotoxic activity, thus maintaining maternal-fetal immune tolerance and ensuring normal pregnancy progression. Fortunately, after active treatment, the patient began immunoglobulin therapy with two courses starting at 16 weeks of gestation. Subsequent fetal follow-ups throughout the pregnancy showed no signs of cardiac abnormalities.

The strength of this case lies in the comprehensive use of multiple therapeutic agents, including corticosteroids, hydroxychloroquine, intravenous immunoglobulin, low-molecular-weight heparin, and aspirin. The combination of thorough imaging and laboratory monitoring demonstrated a systematic and scientific approach to management. In addition, multidisciplinary collaboration among the departments of obstetrics, rheumatology, and ultrasonography played a key role in improving diagnostic efficiency and pregnancy outcomes. The patient underwent dynamic follow-up and risk assessment throughout the entire pregnancy, providing valuable experience for the management of high-risk pregnant women.

However, this case also has certain limitations. First, as a single case report, the conclusions drawn lack support from large-sample or controlled studies and therefore cannot be generalized to all similar patients. Second, although multiple immunosuppressive and anticoagulant therapies were used, the specific effects and optimal combinations of these drugs in improving pregnancy outcomes remain unclear. At present, there is no unified international standard for the treatment of atypical OAPS and pregnant women positive for anti-SSA/SSB antibodies, and the optimal treatment strategies still require further prospective, multicenter studies for validation.

Overall, although atypical OAPS has a low clinical incidence, it poses significant risks due to its potential association with severe adverse pregnancy outcomes, such as fetal intrauterine death, preeclampsia, and fetal growth restriction. Therefore, it requires attention from obstetric professionals. Pregnant women who present with vaginal bleeding or unexplained miscarriage should undergo screening for autoimmune diseases, actively monitor their condition, and seek multidisciplinary consultation, following medical advice to improve pregnancy outcomes and reduce maternal and fetal complications.

## Conclusion

This case report illustrates the successful management of a challenging pregnancy with atypical OAPS complicated by positive anti-SSA/SSB antibodies. It highlights the necessity of proactive intervention in patients who present with a high risk of adverse obstetric outcomes yet do not fulfill the classic diagnostic criteria. Through a combined regimen of immunomodulators (corticosteroids, hydroxychloroquine, and intravenous immunoglobulin) and the timely introduction of anticoagulation therapy (low-molecular-weight heparin and aspirin), we achieved the resolution of a progressively enlarging subchorionic hematoma and ensured maternal-fetal immune tolerance, culminating in a full-term live birth. This favorable outcome underscores the critical importance of risk-based, multidisciplinary management, dynamic laboratory and imaging monitoring, and individualized treatment decisions in complex autoimmune pregnancies. Future prospective studies with larger cohorts are warranted to validate the efficacy of such combined regimens in patients with atypical OAPS and to explore the role of novel biomarkers in early diagnosis and risk stratification.

## Data Availability

The raw data supporting the conclusions of this article will be made available by the authors, without undue reservation.
